# Mental Health and Life Events among United States adolescents with Substance Use Disorders

**DOI:** 10.1192/j.eurpsy.2023.284

**Published:** 2023-07-19

**Authors:** R. Sultan, A. Zhang, F. Levin

**Affiliations:** Child and Adolescent Psychiatry, Columbia University, New York, NY, United States

## Abstract

**Introduction:**

Substance use during adolescence is linked to adverse biopsychosocial events, including poor mental health, cognitive deficits, low academic performance, and delinquency (Deas & Brown J Clin Psych 2006; 67 18; Armstrong & Costello JCCP 2002; 70 1224; Cox et al. JSH 2007; 77 109-115; Chassin JJSU 2008; 165-183). Identifying risks for these events is critical, given they are associated with adverse outcomes in adulthood.

Post-pandemic, rates of adolescent depression and anxiety have more than doubled (Racine et al. JAMA Ped 2021; 175 1142-1150). Adolescents often use substances, most commonly alcohol and cannabis, to manage mental health (Colder et al. JCCP 2019; 87 629).

Cannabis is increasingly viewed by adolescents as safe, while alcohol is viewed negatively (SAMHSA 2021). Non-disordered alcohol use (ND-AU), alcohol use below diagnostic criteria level, has adverse developmental impacts for adolescents, including increased risk-taking behavior and heavy substance use in adulthood (Marshall Alcohol Alcohol. 2014; 49 160-164).

With growing normalization of cannabis use, important questions still remain whether non-disordered cannabis use (ND-CU) among adolescents is linked to adverse life events.

**Objectives:**

Using data from the 2018-2020 National Survey on Drug Use and Health (NSDUH), an annual US representative survey on substance use and mental health, we compared associations among common adolescent substance use and adverse life events.

**Methods:**

Responses from adolescents aged 12-17 (N=32,407) from the 2018-2020 NSDUH were analyzed. Logistic regression was used to evaluate associations between substance use disorder (SUD) diagnoses and adverse adolescent life events. Adjusted odds ratios (aOR) were obtained while controlling for age, sex, and race/ethnicity. Analyses included sampling weights to account for the US population. Adolescents in ND-CU and ND-AU groups were defined by either past-month or past-year use without their respective SUD diagnosis.

**Results:**

Approximately 5% of adolescents had any SUD, and 1.3% had more than one SUD (Table 1). All SUD variables, including cannabis use disorder (CUD) and alcohol use disorder (AUD), were significant for all adverse adolescent life events (Table 2). Adolescents with a SUD were nearly 3 times more likely to have major depression in the past year, 2.5 times more likely to have a C+ or below grade average, and 10 times more likely to be arrested, than controls. These risks increased with more than one SUD (Table 2). Adverse events were similar between ND-CU and ND-AU. (Figure 1).

**Image:**

**Figure:**
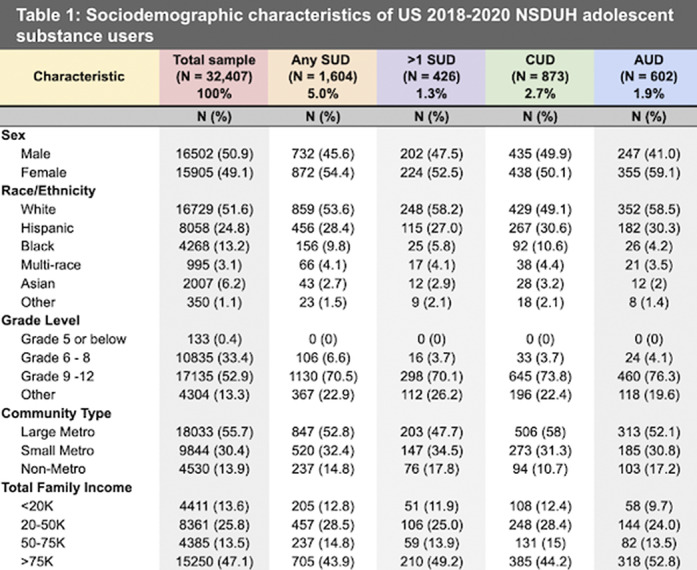

**Image 2:**

**Figure:**
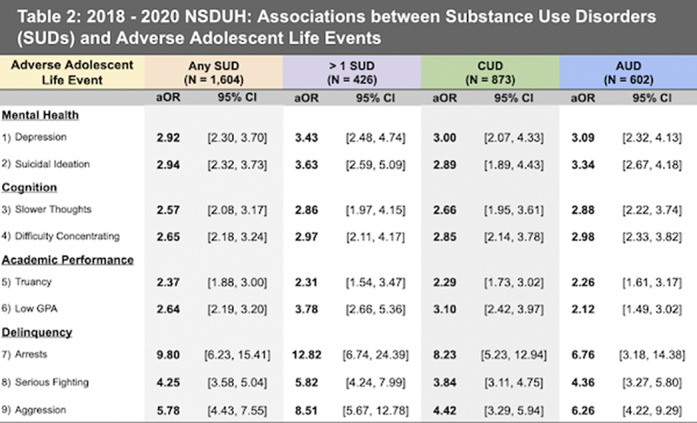

**Image 3:**

**Figure:**
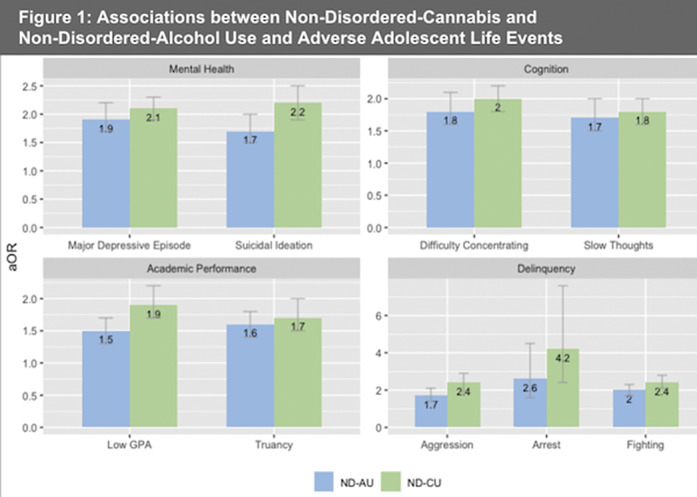

**Conclusions:**

Given the biopsychosocial risks to ND-CU and ND-AU in adolescents, there may be reason to reevaluate whether the DSM adequately captures the population of youth affected by their cannabis and alcohol use. Clinicians can use these nationally representative data to stratify risks and direct to appropriate treatment.

**Disclosure of Interest:**

None Declared

